# Trifarotene: A Current Review and Perspectives in Dermatology

**DOI:** 10.3390/biomedicines9030237

**Published:** 2021-02-26

**Authors:** Terenzio Cosio, Monia Di Prete, Roberta Gaziano, Caterina Lanna, Augusto Orlandi, Paolo Di Francesco, Luca Bianchi, Elena Campione

**Affiliations:** 1 Dermatology Unit, Department of Systems Medicine, University of Rome Tor Vergata, Via Montpellier 1, 00133 Rome, Italy; terenziocosio@gmail.com (T.C.); caterinalanna.cl@gmail.com (C.L.); luca.bianchi@uniroma2.it (L.B.); 2 Anatomic Pathology Unit, Department of Biomedicine and Prevention, University of Rome Tor Vergata, Via Montpellier 1, 00133 Rome, Italy; diprete.monia@gmail.com (M.D.P.); orlandi@uniroma2.it (A.O.); 3 Microbiology Section, Department of Experimental Medicine, University of Rome Tor Vergata, Via Montpellier 1, 00133 Rome, Italy; roberta.gaziano@uniroma2.it (R.G.); difra@uniroma2.it (P.D.F.)

**Keywords:** acne, congenital ichthyosis, T cell lymphoma, RAR-γ agonist, retinoid, trifarotene

## Abstract

Retinoids have numerous applications in inflammatory, dyskeratotic, and oncohematology diseases. Retinoids have now reached the fourth generation, progressively reducing toxicity whilst increasing their efficacy. Trifarotene is a new fourth-generation retinoid with a selective action on RAR-γ. In this review, we reported the trials—both concluded and in progress—including the use of trifarotene in dermatological diseases. Studies were identified by searching electronic databases (MEDLINE, EMBASE, PubMed, Cochrane, Trials.gov) from 2012 to today and reference lists of respective articles. Only articles published in English language were included. Randomized trials evaluating trifarotene tolerability, safety, and efficacy in congenital ichthyosis and acne have demonstrated great results and mild side effects, leading to the approval by the FDA of trifarotene for the treatment of lamellar ichthyosis in 2014, and of acne vulgaris in October 2019. No high-quality randomized clinical trials have evaluated the treatment of primary cutaneous lymphomas with trifarotene. Finally, we are hypothesizing future perspectives in the treatment of non-melanoma skin cancers, fungal infections, photoaging, and hand-foot skin reactions with trifarotene.

## 1. Introduction

Vitamin A (or retinol, a diterpene), a cardinal micronutrient in human metabolism, is a lipophilic molecule composed by isoprene units. As an isoprenoid, it is characterized by a hydrocarbon chain containing an ending hydroxyl. The term “retinoid” concerns both natural and synthetic analogues of vitamin A. In synthetic analogues, such as etretinate, acitretin, or tazarotene, a benzene ring substitutes the cyclohexane. According to the International Union of Pure and Applied Chemistry and the International Union of Biochemistry and Molecular Biology, retinoids are characterized by four isoprene units with a head-to-tail structure [1].

**Figure 1:**
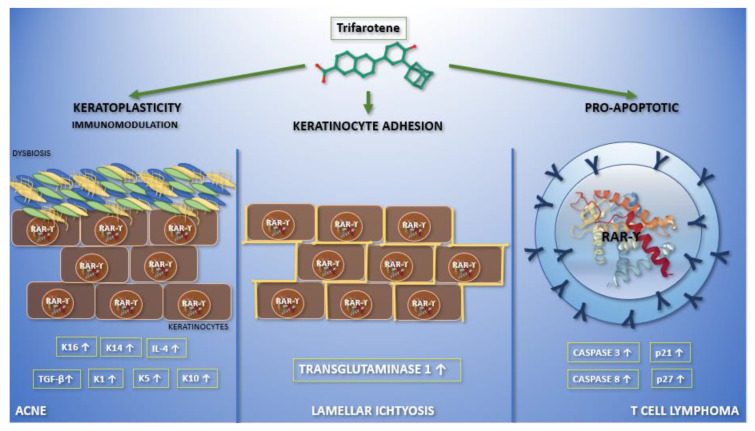
Graphic abstract representing the current applications of trifarotene in dermatological clinical trials and molecular pathways. In the left section, the action of trifarotene in acne is reported. It performs both immunoregulatory—leading to an increase in the expression of transforming growth factor-β and interleukin-4—and keratoplastic functions, increasing the expression of keratins K1, K5, K10, K14, and K16. Moreover, it seems that trifarotene, like other retinoids, may have a role in modulating skin microbiota. Finally, trifarotene weakens hemidesmosomes, interfering with cell adhesion. The migration of keratinocytes, caused by the drug, mediates its comedolytic property. The importance of trifarotene in lamellar ichthyosis has been reported in the central section. Trifarotene, by means of RAR-γ, causes an increased expression of transglutaminase 1, promoting keratinocyte cohesion. The rationale for the use of trifarotene in cutaneous T cell lymphomas has been reported in the right section. It seems to promote apoptosis and differentiation, upregulating caspases 3 and 8, p21 and p27.

It is known that vitamin A and its synthetic analogues have a crucial role in modulating some skin functions; in particular, they regulate epidermal keratinization, differentiation, maturation, and proliferation [2]. Due to all these effects, retinoids are largely used in dermato-oncology, both in treatment and chemo-prevention (non-melanoma skin cancers, primary cutaneous T-cell lymphomas), and even in the treatment of cutaneous inflammatory diseases (acne vulgaris, rosacea, melasma, post-inflammatory hyperpigmentation, mycosis) and hyperproliferative conditions (ichtyosis, psoriasis, pityriasis rubra pilaris) [2]. Moreover, they play a central role in protecting the skin from free radicals damage, as shown by their use also in photoaging. The aim of this review is to highlight the current clinical application (Figure 1) of trifarotene and future perspectives in dermatology.

## 2. Mechanism of Action of Vitamin A and Its Analogues

Stimulated by Retinoic Acid 6 (STRA6), the cell surface receptor mediates the uptake of vitamin A from plasma [3]. Intracellular bioavailability is regulated by specific cytoplasmic retinol and cellular retinoic acid-binding proteins, CRBPs and CRABPs, respectively. CRBPs comprise four isoforms, from CRBP-1 to CRBP-4. The first one is the most represented in many tissues. CRABPs comprise two isoforms, CRABP-1 and CRABP-2. CRBPs and CRABPs specifically bind retinol and retinoic acid (RA), respectively. CRABPs may regulate interactions between RA and its nuclear receptors, influencing bioavailable RA concentrations [4]. Retinoids can activate specific genes expression involved in keratinocytes differentiation, proliferation and apoptosis, binding specific nuclear receptors: retinoic acid receptor (RAR) and retinoid X receptor (RXR) [5]. In humans, there are three genes for each receptor (RAR-α, RAR-β, and RAR-γ and RXR-α, RXR-β, and RXR-γ), each one encoding for several isoforms [6]. RARs take steps as heterodimers with RXRs to activate the transcription of target genes, generally modulating factors, before their ligand, all-trans RA (ATRA) [5].

Upon RA binding, the RAR-RXR heterodimer recognizes specific DNA sequences, named retinoic acid response elements (RAREs) [7,8]. Recent data highlighted that RARs activity can be controlled also by phosphorylation [9], and this event is essential for the regulatory potential of RARs. In fact, in the absence of its ligand, RAR-α binds RAREs, acting as a repressor on its target genes [10]. In contrast, RAR-β and RAR-γ isoforms poorly interact with corepressors, since in these receptors, the corepressor-docking site is closed even in the absence of its ligand [11,12]. It is still unclear whether silencing RA target genes transcription is gene or cell specific. One possibility is the recruitment of unconventional co-regulators that inhibit RARs transcriptional activity, binding RAR itself [13]. On the other hand, the ubiquitin-proteasome system could degrade RARs and RXRs determining the conclusion of the transcriptional process [12]. The fact that it has been observed that RARs are ubiquitinated and degraded by the proteasome further supports this hypothesis [14,15].

**Figure 2:**
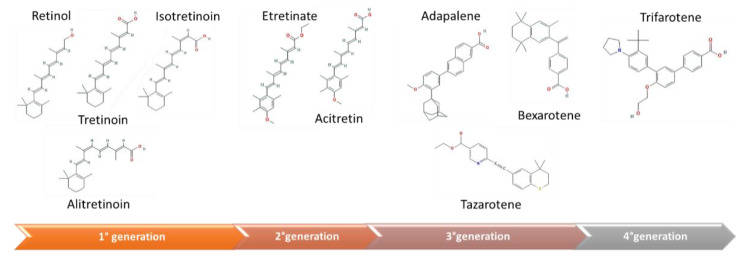
Molecular structure of retinoids divided per generation. The first generation is composed by natural retinoids, obtained modifying polar groups of vitamin A (retinol, tretinoin, isotretinoin, and alitretinoin). The second generation is constituted by monoaromatic compounds, in which a benzene ring replaces the cyclohexene ring (etretinate, and acitretin). The third generation is made up by polyaromatic molecules, resulting from the cyclization of a side chain (adapalene, bexarotene, and tazarotene). Trifarotene is a recently synthetized fourth-generation retinoid, highly specific for skin RAR-γ receptors (References in Supplementary materials).

Retinoids can be classified into three generations, in respect of molecular structures and properties (Figure 2):

1. The first generation is composed by natural retinoids, obtained modifying polar groups of vitamin A, which do not act selectively: retinol and its metabolites, such as retinal, tretinoin, isotretinoin, and alitretinoin;
2. The second generation is constituted by monoaromatic retinoids, synthetic compounds where a benzene ring replaces the cyclohexene ring: etretinate, and acitretin;
3. The third generation is made up by polyaromatic retinoids, resulting from cyclization of the side chain and characterized by a selective activity towards receptor: adapalene, tazarotene, and bexarotene [16].

In the skin, RAR-γ is the most represented isoform [17], sustaining the rational use of topical RAR-γ agonists. Therefore, Thoreau and colleagues described the structure of RAR-γ ligand binding domain, permitting the design of a novel triaryl series of agonists, which was optimized and ultimately led to trifarotene, a new fourth-generation topical retinoid [18]. Aubert and colleagues have described the preclinical pharmacological features of trifarotene [19], which has been approved for the treatment of lamellar ichthyosis, in 2014, and acne vulgaris, in October 2019, by the FDA and is currently undergoing approval even by the EMA [20,21]. Trifarotene is a strong and selective agonist of RAR-γ, with lower activity on RAR-β and RAR-α (16- and 65-fold, respectively), and has no activity on RXRs [19]. The binding of trifarotene on RAR-γ results in the dimerization of the receptor, leading to attach specific RAREs of retinoid-responsible genes. Downstream gene expression alterations are the principal way through which trifarotene exerts its anti-inflammatory, comedolytic, and depigmenting actions [21].

Trifarotene influences three different pathways, identified by a large-scale gene expression analysis:

1) Skin hydration: trifarotene induces skin peptidyl arginine deiminase 1 and aquaporin-3 channels, and, therefore, influences skin barrier functions;
2) Cell adhesion: trifarotene weakens hemidesmosomes, reducing intercellular adhesion. The minor cohesion among keratinocytes explains its comedolytic properties;
3) Proteolysis: trifarotene downregulates matrix metalloproteinases (MMPs), which act as proteolytic enzymes on elastin and collagen, thus improving skin texture [19,22].

Ex-vivo pharmacokinetic models on trifarotene proved the high stability of the compound, with a half-life of >24 h. Despite this, it is quickly metabolized by hepatic microsomal enzymes, with a half-life of minutes, compared to tazarotenic acid, which has a 10-fold higher stability in hepatic microsomes. This is a predictor of a favorable safety profile. The metabolism of trifarotene is catalyzed primarily by cytochrome (CY) P2C9, CYP3A4, CYP2C8, and, to a lesser extent, by CYP2B6 [18].

## 3. Methods and Study Design

### 3.1. Search Strategy

We performed a comprehensive search in the following databases from 2012 to 2020: Cochrane Central Register of Controlled Trials; MEDLINE; Embase; US National Institutes of Health Ongoing Trials Register; NIHR Clinical Research Network Portfolio Database; and the World Health Organization International Clinical Trials Registry Platform. We studied reference lists and published systematic review articles. We used the following keywords, separately and in a combination: “trifarotene”, “CD5789”, “skin”, “dermatology”. Only English language articles were included in the searches. The search was restricted to studies on humans. Forward citation searching of the reference lists of the original studies and review articles was also conducted.

### 3.2. Inclusion Criteria

All the studies investigating the use of trifarotene in skin disorders were examined. If a study included trifarotene with other drugs, only trifarotene frame was analyzed. All human studies were included with no restrictions on age, sex, ethnicity, or type of study. Case reports and case series were included if they described the use of trifarotene in diseases not present in reviews or trials.

### 3.3. Exclusion Criteria

The target intervention excluded the analyses of other pathologies out of the dermatological field, animal studies, and non-English language articles.

### 3.4. Search Results

Eleven trials including trifarotene were identified by this quantitative research. Four resulted completed at the time of this Review, all regarding acne vulgaris, the others have no results published on Trial.gov or Pubmed. Despite the FDA’s 2014 approval, of trifarotene for the treatment of lamellar ichtyosis, no data on large-scale trial are currently available. At present, only one trial has evaluated the safety and tolerability of trifarotene in early-stage primary cutaneous T-cell lymphoma patients, but no results have been published yet.

## 4. Trifarotene Properties and Current Applications in Dermatology

### 4.1. RAR-γ Selectivity

RAR-γ selective retinoid derivatives such as trifarotene, are being investigated as topical agents, which are expected to offer a more favorable clinical profile compared to the dual RARβ/γ drugs currently used in the clinical practice. Trifarotene is a potent and selective agonist of RAR-γ, with significantly less activity on RAR-β and RAR-α (16- and 65-fold lower, respectively), and has no activity on RXRs [19]. Although tazarotene has high affinity for all retinoic acid receptor isoforms, its affinity is roughly 5–8 times higher for the β isoform [23]. Adapalene is selective for the β and γ RARs over the α isoform [24]. Selectivity is a key feature in trifarotene, which allows the action on the keratinocytes, as a primary target, and reduces systemic adverse effects (Table 1). We know, from the Human Protein Atlas project, that RAR-γ m-RNA has the maximum expression in the skin (mean reads per kb per million reads placed 47.135 ± 3.294, compared to RAR-β 0.272 ± 0.138, count 3,210,032 for RAR-γ vs. 21,114 for RAR-β; Figure S1) [25], sustaining the rational use of a topical RAR-γ agonist.

### 4.2. Trifarotene Safety and Tolerability

Trifarotene is metabolized in vitro by CYP2C9, CYP3A4, CYP2C8, and, to a lesser extent, by CYP2B6, and excreted in the feces [18]. Systemic exposures in mice, following both topical and oral administration, were up to 1642 times higher than those seen in humans at the maximal recommended human dose, and these systemic concentrations did not result in observed carcinogenicity. Trifarotene does not seem to carry any risk of carcinogenesis when used at standard doses [26,27]. Data regarding overdosage of trifarotene are not available. Patients exposed to photosensitising agents may have an increased risk of a phototoxic skin reaction, especially severe sunburn, during the use of aminolevulinic acid [28]. Concomitant use of retinoids and keratolytic or topical astringents may result in excessive irritation and/or drying, and patients may experience erythema, scaling, dryness, and stinging/burning [26,27]. Despite these local adverse effects and photosensitization, trifarotene is safer than other retinoids due to its hepatic instability and degradation. Current studies confirm that trifarotene 50 µg/g cream is systemically well tolerated and safe when applied under maximized conditions in adults and pediatric acne patients, including patients with severe acne [27]. Considering that trifarotene belongs to the class of retinoids and is intended for use even in women in their fertile age, further studies are needed to exclude any potential teratogenic effect. Currently, clinical pharmacological data demonstrate that trifarotene 50 µg/g cream—the to-be-marketed formulation—generates low systemic absorption when applied daily under maximal use conditions [27]. Furthermore, due to its RAR-γ selectivity, it could be hypothesized that trifarotene is safer than other retinoids in pregnancy as the placenta presents a lower expression of RAR-γ (4.739 ± 0.399), with consequent minor absorption of the drug compared to other topical retinoids [25].

### 4.3. Current Applications in Dermatology

Herein we illustrate the rational use of trifarotene in skin diseases.

#### 4.3.1. Acne Vulgaris

Acne vulgaris is a chronic inflammatory process of the pilo-sebaceous unit. Overproduction and abnormal cohesiveness of desquamated epithelial cells leads to their retention within the hair follicle, with subsequent obstruction of the ostium [29]. Pro-inflammatory mediators, such as interleukin (IL)-1 and tumor necrosis factor (TNF)-α, are produced by keratinocytes, activated in response to epithelium disruption, caused by accumulating sebum. The clinical course is characterized by subsequent phases of remission and recurrence. In some individuals, acne may persist for decades and leave scars. The association of acne with depression, anxiety, and reduced quality of life is well documented [30]. Successful treatments may produce a significant improvement in self-esteem [31]. Both the American Academy of Dermatology (AAD) and the European Dermatology Forum guidelines agreed that retinoids play a crucial role in the treatment of acne [32,33]. The AAD guidelines declare that “retinoids are the core of topical therapy for acne because they are comedolytic, resolve the precursor microcomedone lesion, and are anti-inflammatory”; additionally, they “allow for the maintenance of clearance” [33].

Retinoids promote keratinocytes differentiation and diminish their proliferation, modulating desquamation [34]. Topical retinoids block critical inflammatory pathways triggered in acne, such as leukocyte migration, Toll-like receptors activation, and the Activator Protein 1 pathway [35].

Trifarotene is the latest new retinoid approved for the treatment of acne by the FDA in over 20 years. Topical administration is demonstrated to be safe, well-tolerated, and more effective than vehicle in reducing both non-inflammatory and inflammatory lesions in acne of the face and trunk [36]. We evaluated two phase III, double-blind, randomized, vehicle-controlled, 12-week studies in which trifarotene 50 μg/g cream was administered once-daily versus vehicle in subjects aged nine or older (Table 2). In PERFECT 1 trial, a total of 1208 patients were recruited and divided in two arms: 612 were treated with trifarotene and 596 received placebo. In PERFECT 2 trial, 1212 patients were enrolled and randomized in two treatments: the first group was constituted by 602 patients, who received trifarotene, while the placebo group included 610 patients [36]. For both studies, the primary endpoints were the rate of face lesions resolution, determined by Investigator’s Global Assessment (IGA), and change in absolute number of face non-inflammatory and inflammatory lesions from baseline to week 12. Secondary endpoints were the rate of trunk lesions resolution, according to Physician’s Global Assessment (PGA), and change in the absolute number of trunk non-inflammatory and inflammatory lesions from baseline to week 12 [36]. The safety profile was assessed in terms of adverse events (AEs), local tolerability, vital signs, and routine laboratory tests. In both studies, at week 12, primary and secondary endpoints were reached and the results were highly significant (*p* < 0.001) in favour of trifarotene, demonstrating its safety, efficacy, and tolerability in acne treatment of both face and trunk [36]. Blume-Peytavi et al. obtained comparable results in a multicentre, open-label study considering 453 patients (Table 2). They reached a global success rate of 57.9% after 52-week treatment with trifarotene 50 μg/g cream in both face and trunk acne, evaluated by IGA and PGA, as primary and secondary endpoints respectively [37]. Adverse events were observed in 12.6% of patients, especially during the first trimester of treatment; they were mild-to-moderate in severity and included itching, erythema, burning, stinging, and dryness. Rare severe AEs consisted in sunburn sensation, allergic dermatitis, pain, and cutaneous erosion in the site of application, resulting in treatment discontinuation in 1.9% participants [37]. All subjects reported an improvement in the quality-of-life index at week 52 from baseline [37].

Johnson et al. reported a series of three subjects with moderate face and trunk acne treated with trifarotene 50 µg/g cream for 12 weeks, then evaluated through questionnaires. A reduction in inflammatory lesions was reported. The reduction in the lesions on the face and trunk was 90% and 47% for inflammatory and non-inflammatory lesions, respectively, in the first subject. The second one achieved a 20% reduction in inflammatory lesions and 22% reduction in non-inflammatory lesions both on the face and trunk. In the last subject, a reduction of 66% and 34% was observed in inflammatory and non-inflammatory lesions, respectively [38].

Many trials are still ongoing or have not yet produced results on the use of trifarotene in acne vulgaris (Table 3).

Retinoids can indirectly affect skin microbes, blocking the essential nutrients supply and stabilizing immune system hyperreactivity [39,40]. In this light, the importance of retinoid therapy in acne is due to the indirect effect on microbiota, which opens new frontiers in terms of therapies. The suppressing role of retinoids in sebum production by the sebaceous glands both in vitro and in vivo has also been highlighted [41,42]. In fact, while low levels of RA are important for sebaceous gland function, excessive RA synthesis within the sebaceous gland could lead to atrophy of the gland, and reduced sebum production [43]. Finzi et al., using a specific oligonucleotide for RAR-γ cDNA isoform 1 (RAR-γ 1), discovered that RAR-γ 1 mRNA was localized in all epidermal layers, outer root sheath of hair follicles, follicular hair bulbs, eccrine, and sebaceous glands [44]. They suggested that the presence of RAR-γ in sebaceous glands could mediate the ability of isotretinoin to suppress sebum production in nodulocystic acne [44]. Trifarotene, as a selective RAR-γ agonist, may be effective in acne vulgaris treatment, among the other mechanism, also inhibiting sebaceous glands function.

#### 4.3.2. Autosomal Recessive Congenital Ichthyosis

Autosomal recessive congenital ichthyosis (ARCI) is a heterogeneous family of congenital diseases of keratinization linked to generalized hyperkeratosis, often accompanied by erythroderma. ARCI is infrequent, with 1 in 200,000 births incidence. The most frequent phenotypic subtypes include lamellar ichthyosis, congenital ichthyosiform erythroderma, and harlequin ichthyosis. ARCI is also genetically heterogeneous—with at least nine different genes responsible for the most common forms—but approximately 30% of cases are explained by TGM1 mutations, the gene encoding transglutaminase 1, involved in the development of the cornified envelope [45]. ATP-binding cassette (ABC) transporter, family 12 (ABCA12) facilitates lipids delivery to the lamellar bodies (LB) in keratinocytes, which is critical for the barrier function permeability. Recently, ABCA12 mutations were described in harlequin and lamellar ichthyosis. Jang et al. observed that peroxisome proliferators-activated receptors (PPARs) and liver X receptor (LXR) activation improve epidermal barrier permeability, stimulating keratinocyte differentiation, lipid synthesis, and LB formation/secretion [45]. Both PPAR-γ and LXR activators were reported to stimulate, in a dose- and time-dependent manner, ABCA12 mRNA expression in cultured human keratinocytes (CHKs). Increased ABCA12 mRNA levels are accompanied by an increase in ABCA12 protein synthesis. By demonstrating that PPAR and LXR activators increase ABCA12 expression, they provided an additional mechanism through which PPAR and LXR activators promote epidermal barrier permeability [45]. In contrast, ABCA12 expression is not altered by the activators of PPAR-α, RAR, or RXR. 

The efficacy of retinoids to treat this disease has been known for some time [46]. Virtanen et al. assessed phenotypic/genotypic correlations in patients with epidermolytic hyperkeratosis and the impact of retinoid therapy on keratin expression [47]. Thirteen patients from ten families with generalized disease and two subjects with sporadic disease with naevoid lesions were studied. Oral acitretin (5–25 mg/d) or topical tretinoin/tazarotene were effective in five of six patients with keratin 10 mutations, while none of those with keratin 1 mutations showed any advantage [47,48].

As topical trifarotene was demonstrated to be safe and well-tolerated, in 2014, the FDA grated it with Orphan Drug Designation for the treatment of congenital ichthyosis [20]. We evaluated a phase II, randomized, multicenter, double-blind, vehicle-controlled, 12-week topical trifarotene treatment for moderate to severe autosomal recessive lamellar ichthyosis in adults (≥18 years old) and adolescents (ages 12–17 years, inclusive), followed by a 12-week open-label extension (Table 4). Adults (cohort A) and adults and adolescents (cohort B) were randomized in a double-blind fashion to active therapy or vehicle and were treated twice weekly for 12 weeks. Patients who completed this step of the study without safety issues were selected to enter a 12-week, open-label extension of the trial. All subjects, both adults and adolescents, were randomized 1:1:1 and treated twice weekly for up to a further 12 weeks. The results of the study will be available probably by the end of the year [49].

#### 4.3.3. Primary Cutaneous T-Cell Lymphoma

Cutaneous T-cell lymphomas (CTCLs) are primary skin lymphoproliferative disorders, deriving from mature T cells. CTCLs could be indolent or aggressive processes and this great heterogeneity explains the difficulties in clinical management. Sixty percent of all CTCL cases is represented by mycosis fungoides, while less than 5% consists of Sézary syndrome, a more aggressive form [50,51,52,53,54,55,56].

Retinoids display a crucial role among WHO and EORTC recommended therapies for CTCLs. They have been used in lymphoma of every stage for over three decades [55]. In vitro studies demonstrated that retinoids modulate keratinocytes proliferation and differentiation, and regulate skin mononuclear inflammatory infiltrate, but also induce apoptosis and DNA fragmentation in T-cell lines [57]. 

Sidell et al. observed that ATRA could increase IL-2Rα expression in human thymocytes, increasing steady-state mRNA levels [58]. Gorgun and Foss confirmed these results exploring the effect of ATRA, bexarotene, and alitretinoin (which binds both RAR and RXR) on human T-cell and B-cell leukaemia cell lines. All three molecules induced both α and β subunits of the IL-2R upregulation. Analogous discoveries were observed in the same study with Sézary cells and B-cell lymphocytic leukaemia cells [59]. Many studies have focused on systemic retinoids, but also the efficacy and tolerability of topical retinoids in CTCLs have been tested. Skin directed therapies have an important role in the treatment of early stage CTCLs, in particular MF, as well as in managing symptoms and improving quality of life at all stages. Retinoids found a place in this scenario due to their immunomodulatory and pro-apoptotic actions. Topical bexarotene 1% gel is approved by the FDA for stage IA and IB persistent or refractory CTCLs. It has been reported to cause apoptosis in CTCL cell lines [60,61]. In 2016, ten patients with early stage CTCL/MF were treated with tazarotene 0.1% cream in monotherapy on target lesions every other day for 2 weeks, then once daily for 6 months. Six patients had clinical resolution of the target lesion, within a mean time of 3.8 months. The vast majority (70%) reported itching, burning, erythema, and desquamation, whilst two patients withdrew from study [61,62]. Nowadays, no high-quality randomized clinical trials evaluating the treatment of primary CTCL with trifarotene has been completed. At present, only one phase I trial evaluating safety and tolerability of trifarotene in patients with early stage CTCL has been reported in literature, though no results are currently available (Table 5) [63].

## 5. Perspectives

### 5.1. Non-Melanoma Skin Cancer

**Figure 3:**
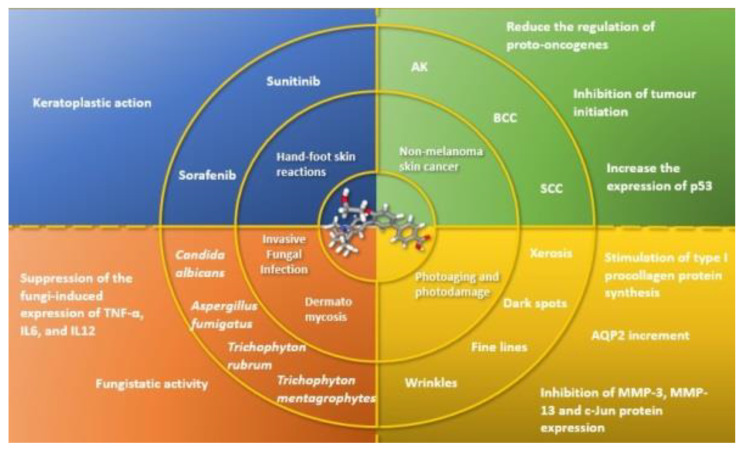
Schematic representation of the perspective applications of trifarotene with a brief explanation of the putative molecular pathways.

Retinoids are largely used for the prevention and treatment of non-melanoma skin cancers (NMSCs). Since the 1920s, when vitamin A deficiency in rats paved the way to investigate its relationship with cancer, retinoids have played a pivotal role in the oncology field [64]. Biochemical studies in the 1970s and 1980s suggested that a relative deficiency of retinoids could be associated with epithelial cancers [65]. To date, retinoids have demonstrated anti-apoptotic and anti-proliferative properties; indeed, they are able to regulate the differentiation and growth of keratinocytes, inhibit tumor initiation, reduce regulation of proto-oncogenes, increase the expression of p53 and pro-apoptotic caspases, and sensitize keratinocytes to apoptosis [66]. In murine models of skin carcinogenesis, retinoids target the B-Raf/Mek/Erk signalling pathway [67]. Moreover, retinoids have shown antioxidant properties, reducing the number of sunburn cells, and acting against the human papillomavirus, which is considered a co-carcinogen [68,69]. In NMSCs, retinoids have been used to treat precancerous lesions, such as actinic keratoses (AKs), as they inhibit the development of invasive cancer [70]. Bollag et al., in their case series, observed a 50% reduction of AKs on arms and hands with topical application of tretinoin 0.1% and 0.3%, respectively [71]. In basal cell carcinoma (BCC), tazarotene induced a concentration-dependent increase in RAR-β and bax, which was associated with a greater rate of apoptosis and growth inhibition compared to squamous cell tumors. Orlandi et al. reported convincing evidence that tazarotene induces BCC regression possibly by synergistic RAR-β-dependent anti-proliferative and pro-apoptotic pathways activation [72,73]. Nijsten et al. reported a decreased squamous cell carcinoma (SCC) risk using systemic retinoids in psoralen-treated patients [74]. Kim et al. evaluated a cohort from 1984 to 2012 and prospectively examined intake of vitamin A and carotenoids and SCC risk in the Nurses’ Health Study and the Health Professionals Follow-up Study. They found a decreased risk of incident SCC related to high intake of dietary vitamin A [75]. As the other compounds from the retinoids family, we may hypothesize that trifarotene could be used as a preventive local therapy in cancerization and as a target therapy for overt NMSCs (Figure 3). Trifarotene can be used alone or combined with other active compounds, working on different pathways of the carcinogenesis, such as nicotinamide or piroxicam [76,77]. Further studies are needed in this promising field.

### 5.2. Invasive Fungal Infection (IFI)

Since ATRA has been used in acute promyelocytic leukemia treatment, a lower incidence of total episodes of fungemia has been reported in these patients [78]. Starting from these observations, retinoids have been investigated as potential fungistatic agents [79]. In vitro ATRA 0.5–1 mM fungistatic effect was proved on Candida albicans and Aspergillus fumigatus by Campione et al. From this study, ATRA paved the way for its systemic use against these opportunistic agents [80]. ATRA stimulates both the adaptive and innate immune system, especially the monocyte-mediate immune response [78,81]. Klassert et al. provided evidence of ATRA immunomodulatory effect on human monocytes during Candida albicans infections, suppressing Candida-induced TNF-α, IL-6, and IL-12 production at both transcriptional and post-translational levels [82]. In 2020, Campione et al. performed in vitro experiments to assess ATRA efficacy, associated with classical antifungal drugs, on Aspergillus in a rat model. In silico studies, used to clarify its mechanism of action, showed the strong fungistatic activity of ATRA (0.5 and 1 mM) on Aspergillus cultures, inhibiting fungal Hsp90 expression and Hsp90-related genes and enhancing macrophagic phagocytosis of conidia [83].

From these studies, trifarotene could pave the way for fourth generation retinoids as agents in respiratory and oesophageal mycosis, due to their high selectivity for these districts, with consequent reduced adverse effects (Figure 3). Oesophagus RAR-γ mean reads per kilobase per million of 31.959 ± 6.555 place oesophagus just after the skin for the expression of RAR-γ, as already previously reported. The lungs—another frequent target of mycosis, such as aspergillosis especially in immunocompromised patients—show an expression of RAR-γ mean reads per kilobase per million of 6.541 ± 1.311, suggesting the rationale for a selective RAR-γ-agent, such as trifarotene [25]. Supposing a formulation for systemic administration of trifarotene is out of the scope of this paper. However, it is possible to hypothesize its local application to effectively fight respiratory and esophageal mycosis, although further research is required.

### 5.3. Skin and Nail Mycosis

Onychomycosis is a fungal infection of the fingernails that may involve any component of the nail unit, including the matrix, bed, or plate. Distal and lateral onychomycoses are the most frequent forms of onychomycosis, causing subungual hyperkeratosis that usually limits local penetration of antimycotic drugs. The involved pathogens are dermatophytes, yeasts (*Candida* spp.), and non-dermatophytes [84]. Dermatophytes, such as Trichophyton rubrum and Trichophyton mentagrophytes, are the most common agents of onychomycosis, accounting for 50–90% of cases. Onychomycosis is usually treated with topical or systemic antifungals [85]. Local treatment requires strong patient adherence as the treatment time is generally long. Moreover, onychomycosis-related subungual hyperkeratosis is generally thick, an aspect that limits the penetration of the antimycotic drug. Consequently, long-term treatments can discourage or induce withdrawal before healing is achieved. Campione et al., from in vitro studies, reported that tazarotene can inhibit the growth of Trichophyton rubrum, Trichophyton mentagrophytes, and Candida albicans. In the same study, they demonstrated that topical 0.1% tazarotene gel is an effective in vivo treatment for distal and lateral onychomycosis [79]. Since the contribution of the inflammatory response is quite limited in the affected nail, the beneficial effects of retinoids in this field derive from a direct fungistatic activity. From these results, trifarotene could pave the way for fourth generation retinoids as active agents against skin and nail mycosis due to their high selectivity for RAR-γ, with consequent reduced adverse effects, but also actions on the immune system (Figure 3). As previously described, retinoids are immunomodulators, thus, given that the nail unit is an immunological niche, a balance in pro-inflammatory and anti-inflammatory cytokines is paramount [86].

### 5.4. Photoaging

In the 1980s, Kligman et al. suggested for the first time to use retinoids in the treatment of photo-damaged skin [87]. However, the anti-photoaging mechanism of retinoids was elucidated some decades later by Li et al., who demonstrated that ATRA improves skin conditions in UV-induced damage, increasing the collagen content through the RAR pathway, stimulating type I procollagen protein synthesis, and inhibiting MMP-3, MMP-13 and c-Jun protein expression [88]. Rusu et al. reported the efficacy of adapalene in the treatment of photoaging, apart from its utility in several dermatological diseases, as acne vulgaris [89]. In a recent study, Campione et al., demonstrated the clinical efficacy of retinoic acid (0.02%) in photoaging. Twelve women with moderate-severe skin aging (Glogau score ≥ 3) were enrolled and evaluated after 4 weeks of treatment. At baseline, the Glogau score was 3.4 ± 0.5. It then decreased significantly at week 4 (*p* = 0.0001; ANOVA test) to 2.7 ± 0.6. Significant reductions of dark spots (−40%) and severity of wrinkles (−12%) were observed at week 4 compared to baseline. At the end of the study, ultrastructural analyses performed by reflectance confocal microscopy also highlighted significant improvements, with the recovery of the polygonal keratinocytes pattern as the main observed aspect [90]. Trifarotene, acting as a RAR-γ agonist, could activate the same pathways induced by ATRA, eliciting antiaging activity (Figure 3). Further studies are needed in this field, which is currently quite unexplored.

### 5.5. Hand-Foot Skin Reaction

Targeted therapy has improved the oncological management and survival of patients. Sorafenib and sunitinib are two novel, small-molecule multikinase inhibitors that have shown promising results in the inhibition of tumor cell angiogenesis and proliferation, both in vitro and in vivo [91]. Despite their higher specificity, when compared to standard chemotherapy, the activity of these agents is not limited to tumor cells. A variety of adverse effects have been reported, including diarrhea, hypertension, and nausea. Among them, the most notable, cutaneous toxic effect, known as hand-foot skin reaction (HFSR), represents 20–30%. HFSR consists in a diffused painful oedema and redness of palms and soles due to epidermal keratinocyte apoptosis, dyskeratosis, and vacuolar degeneration. Lacouture et al. reported the efficacy of tazarotene against HFSR occurring in patients treated with sorafenib and sunitinib [92]. A phase II completed trial has evaluated the usage of tazarotene 0.1% cream in HFSR of patients treated with sorafenib. Tazarotene 0.1% cream has been applied twice per day on the affected areas, with no adverse effects. Results demonstrated a 9.5 reduction (12.0) in Skindex-16 Total Score [93]. Nowadays, a phase II randomized Double-Blind Trial is going to evaluate the effectiveness of topical tazarotene 0.1% gel to prevent regorafenib-induced HFSR [94]. As previously reported, trifarotene, acting as selective RAR-γ agent, could play a keratoplastic role in reducing HFSR and normalizing keratinization (Figure 3).

## 6. Conclusions

Retinoids are largely applied in cutaneous inflammatory, dyskeratotic, and infectious diseases, besides oncohematology. In this review, we have focused our attention particularly on trifarotene, a new fourth-generation retinoid with a selective action on RAR-γ. Trifarotene has been tested for the treatment of acne and congenital ichthyosis, demonstrating its safety and tolerability. No high-quality randomized clinical trials are currently evaluating the treatment of primary cutaneous lymphomas with trifarotene. Differently from other retinoids, trifarotene acts selectively on RAR-γ reducing RAR-β adverse effects, and offer a more favorable clinical profile compared to the drugs with dual action on both RAR-β and RAR-γ, such as tretinoin and derivatives. As RAR-γ is much more abundant than the other retinoids receptors in the skin, this could be the rationale for increasing studies on trifarotene usage in skin disorders, as already seen with previous generation retinoids.

## Figures and Tables

**Table 1:** The table shows the different drugs capable of interacting with the different RAR isoforms. Of all the drugs reported, only trifarotene and palovarotene are selective for RAR-γ. Currently, palovarotene is used in the treatment of progressive ossifying fibrodysplasia, while trifarotene is used in lamellar ichthyosis and acne. The importance of receptor selectivity is confirmed by the reduction of adverse effects due to the action on the other receptors.

Receptor		RAR-α	RAR-β	RAR-γ
Tissue Expression		Lung, Spleen, Gallbladder	Placenta, Prostate, Urinary Bladder, Kidney, Heart	Skin
**Drug**	Tazarotene	☑	☑	☑
	Tretinoin	☑	☑	☑
	Trifarotene	X	X	☑
	Adapalene	X	☑	☑
	Alitetrinoin	☑	☑	☑
	Tamibarotene	☑	X	X
	Palovarotene	X	X	☑

**Table 2:** Completed clinical trials with results evaluating trifarotene in acne vulgaris. Abbreviations: IGA: Investigator’s global assessment; PGA: Physician’s global assessment.

Official Title on ClinicalTrials.gov or Publication Title (NCT Number and Status)	Phase; Evaluation Time; Sample Size	Endpoints and Results
A Multi-Centre, Randomized, Double-Blind, Parallel-Group Vehicle Controlled Study to Compare The Efficacy And Safety Of CD5789 (Trifarotene) 50 μg/g Cream Versus Vehicle Cream In Subjects With Acne Vulgaris (NCT02566369; Completed) [36]	III; 12 weeks; 1208 patients, randomized, parallel assignment	IGA: trifarotene arm 42.6%; placebo arm 25.8%
A Multi-Center, Randomized, Double-Blind, Parallel-Group Vehicle Controlled Study To Compare The Efficacy And Safety Of CD5789 (Trifarotene) 50 µg/g Cream Versus Vehicle Cream In Subjects With Acne Vulgaris (NCT02556788; Completed) [36]	III; 12 weeks; 1212 patients, randomized, parallel assignment	IGA: trifarotene arm 29.4%; placebo arm 19.5%
A long-term safety and efficacy study of cd5789 (trifarotene) 50 µg/g cream in subjects with acne vulgaris (NCT02189629; Completed) [37]	III; 52 weeks; 453 patients, single group assignment	- Primary: IGA 65.1% - Secondary: PGA 66.9%

**Table 3:** Clinical trials evaluating trifarotene in acne vulgaris with no results reported yet. Abbreviations: IGA: Investigator’s global assessment.

Official Title on ClinicalTrials.gov or Publication Title (NCT Number and Status)	Phase; Sample Size	Drugs Evaluated	Endpoints and Results
A Randomized, Multi-centre, Investigator-blind, Vehicle- and Active-controlled, Phase 2 Study to Assess the Efficacy and Safety of Different Concentrations of CD5789 Cream Applied Once Daily in Subjects With Moderate to Severe Acne Vulgaris (NCT01616654;Completed)	II; 304 patients, randomized, parallel assignment	CD5789 25 µg/g cream; CD5789 50 µg/g cream; CD5789 100 µg/g cream; tazarotene 0.1% gel; vehicle cream	- Endpoints: (1) success rate (IGA); (2) absolute change in total lesion counts; (3) percentage ghange in total lesion counts - Results: not yet reported
A Multi-Centre Study to Evaluate Subject Reported Outcomes with Use of Trifarotene 50 μg/g Cream in the Treatment of Moderate Facial and Truncal Acne Vulgaris (NCT03915860 Active, non-recruiting)	III; 40 patients, single group assignment, open label	Trifarotene 50 μg/g cream	- Endpoint: success rate (IGA) score of 1 or 0 and at least a 2-grade improvement - Resuls: not yet reported
A Multi-Centre, Randomized, Double-Blind, Placebo Controlled Study to Compare Efficacy and Safety of Trifarotene (CD5789) Cream When Used with an Oral Antibiotic for the Treatment of Severe Acne Vulgaris (NCT04451330; Not recruiting)	IV; 198 patients, randomized, parallel assignment	Trifarotene cream; Doxycycline hyclate; Trifarotene Vehicle; Doxycycline Placebo	- Primary endpoint: change in facial total lesion counts (inflammatory and non-inflammatory) - Secondary endpoints: (1) change in facial inflammatory lesions counts; (2) change in facial non-inflammatory lesions count - Results: not yet reported

**Table 4:** Clinical trial evaluating trifarotene in lamellar ichtyosis with no results reported yet.

Official Title on ClinicalTrials.gov or Publication Title (NCT Number and Status)	Phase; Sample Size	Endpoints and Results
A Phase 2 Randomized, Multicenter, Doubleblind, Vehicle Controlled, 12 Week, Safety, Efficacy & Systemic Exposure Study Followed by a 12 Week Open-label Extension of CD5789 in Adults and Adolescents With Autosomal Recessive Ichthyosis With Lamellar Scale (NCT03738800; Recruiting) [49]	II; 120 patients, randomized, parallel assignment	- Primary endpoint: successful resolution of lamellar ichtyosis - Secondary endpoints: (1) difference in mean scores using Individual score for roughness; (2) difference in mean scores using Palm Sole Assessment; (3) difference in proportion of subjects with fissures between the active and vehicle groups; (4) Dermatology Life Quality Index; (5) 5-point Visual Index for Ichthyosis Severity - Results: not yet reported

**Table 5:** Clinical trial evaluating trifarotene in cutaneous T-cell lymphomas with no results reported yet.

Official Title on ClinicalTrials.gov or Publication Title (NCT Number and Status)	Phase; Sample Size	Endpoints and Results
Exploratory Study to Evaluate the Safety and Efficacy of CD5789 in Subjects with Early Stage Cutaneous T-Cell Lymphoma (NCT01804335; Completed) [63]	I; 11 patients, single group assignment, open label	- Endpoint: tolerance score of CD5789 0.01% cream - Results: not yet reported

## Supplementary Materials

The following are available online at https://www.mdpi.com/2227-9059/9/3/237/s1, References to Figure 2 (PubChem Compound Summary, Accessed Jan. 12, 2021), and Figure S1: Graphic reports kilobase per million reads placed (PRKM) expression of RAR-β and RAR-γ in skin. (modified from BioProject: PRJEB4337, Analysis date: Wed Apr 4 07:08:55 2018).
