# Supplementary material for: Trifarotene: A Current Review and Perspectives in Dermatology
Source: Biomedicines. 2021 Feb 26;9(3):237. doi: 10.3390/biomedicines9030237 (PMC7996910; doi:10.3390/biomedicines9030237)
Supplement: Supplementary file 1 [file biomedicines-09-00237-s001.pdf]

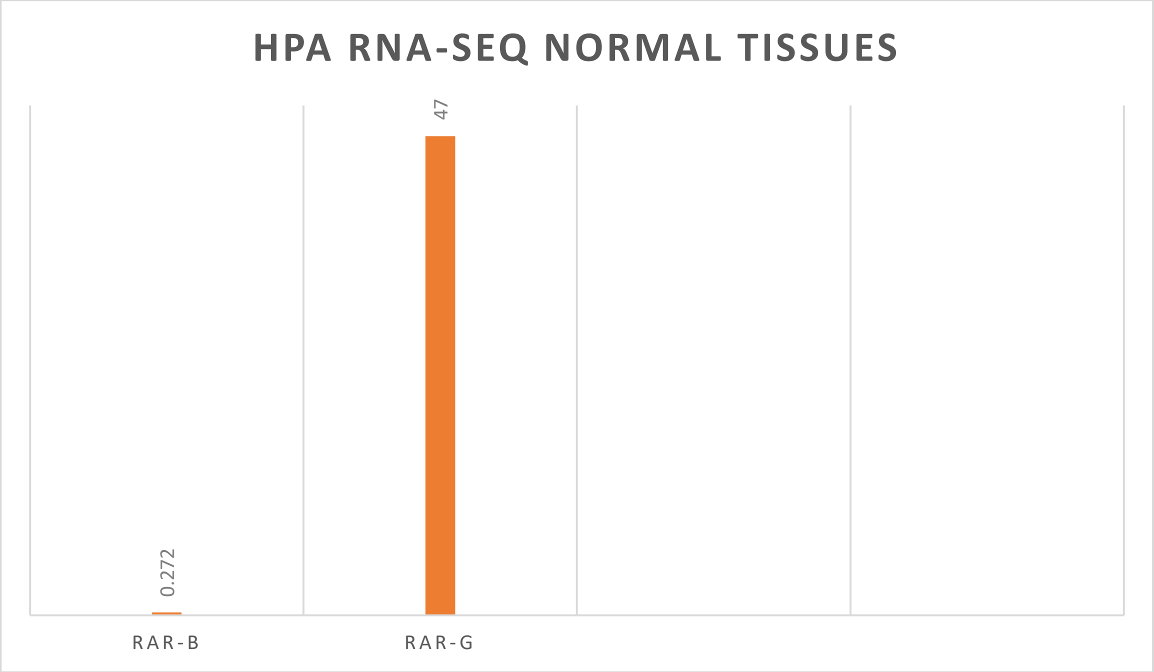

Figure S1. Graphic reports kilobase per million reads placed (PRKM) expression of RAR-β and RAR-γ in skin. (modified from BioProject: PRJEB4337, Analysis date: Wed Apr 4 07:08:55 2018).

References to Figure 2 (PubChem Compound Summary, Accessed Jan. 12, 2021).

1. AMA
National Center for Biotechnology Information. PubChem Compound Summary for CID 445354, Retinol. https://pubchem.ncbi.nlm.nih.gov/compound/Retinol. Accessed Jan. 12, 2021.

2. AMA
National Center for Biotechnology Information. PubChem Compound Summary for CID 444795, Tretinoin. https://pubchem.ncbi.nlm.nih.gov/compound/Tretinoin. Accessed Jan. 12, 2021.

3. AMA
National Center for Biotechnology Information. PubChem Compound Summary for CID 5282379, Isotretinoin. https://pubchem.ncbi.nlm.nih.gov/compound/Isotretinoin. Accessed Jan. 12, 2021.

4. AMA
National Center for Biotechnology Information. PubChem Compound Summary for CID 449171, Alitretinoin. https://pubchem.ncbi.nlm.nih.gov/compound/Alitretinoin. Accessed Jan. 12, 2021.

5. AMA
National Center for Biotechnology Information. PubChem Compound Summary for CID 5282375, Etretinate. https://pubchem.ncbi.nlm.nih.gov/compound/Etretinate. Accessed Jan. 12, 2021.

6. AMA
National Center for Biotechnology Information. PubChem Compound Summary for CID 5284513, Acitretin. https://pubchem.ncbi.nlm.nih.gov/compound/Acitretin. Accessed Jan. 12, 2021.

7. AMA
National Center for Biotechnology Information. PubChem Compound Summary for CID 60164, Adapalene. https://pubchem.ncbi.nlm.nih.gov/compound/Adapalene. Accessed Jan. 12, 2021.

8. AMA
National Center for Biotechnology Information. PubChem Compound Summary for CID 82146, Bexarotene. https://pubchem.ncbi.nlm.nih.gov/compound/Bexarotene. Accessed Jan. 12, 2021.

9. AMA
National Center for Biotechnology Information. PubChem Compound Summary for CID 5381, Tazarotene. https://pubchem.ncbi.nlm.nih.gov/compound/Tazarotene. Accessed Jan. 12, 2021.

10. AMA
National Center for Biotechnology Information. PubChem Compound Summary for CID 11518241, Trifarotene. https://pubchem.ncbi.nlm.nih.gov/compound/Trifarotene. Accessed Jan. 12, 2021.

RARG retinoic acid receptor gamma [Homo sapiens (human)]

Gene ID: 5916, updated on 1-Aug-2020

Project title: HPA RNA-seq normal tissues

Description: RNA-seq was performed of tissue samples from 95 human individuals representing 27 different tissues in order to determine tissue-specificity of all protein-coding genes

BioProject: PRJEB4337

Publication: PMID 24309898

Analysis date: Wed Apr 4 07:08:55 2018

Accessed on 15th September, 2020.
